# Circadian Entrainment in *Arabidopsis* by the Sugar-Responsive Transcription Factor bZIP63

**DOI:** 10.1016/j.cub.2018.05.092

**Published:** 2018-08-20

**Authors:** Alexander Frank, Cleverson C. Matiolli, Américo J.C. Viana, Timothy J. Hearn, Jelena Kusakina, Fiona E. Belbin, David Wells Newman, Aline Yochikawa, Dora L. Cano-Ramirez, Anupama Chembath, Kester Cragg-Barber, Michael J. Haydon, Carlos T. Hotta, Michel Vincentz, Alex A.R. Webb, Antony N. Dodd

**Affiliations:** 1 Department of Plant Sciences, University of Cambridge, Cambridge CB2 3EA, UK; 2 Centro de Biologia Molecular e Engenharia Genética, Departamento de Biologia Vegetal, Instituto de Biologia, Universidade Estadual de Campinas, CEP 13083-875, CP 6010, Campinas, São Paulo, Brazil; 3 School of Biological Sciences, University of Bristol, Bristol BS8 1TQ, UK; 4 Faculty of Biological Sciences, University of Leeds, Leeds LS2 9JT, UK; 5 Universidade Estadual de Campinas, Barão Geraldo, Campinas, São Paulo, Brazil; 6 School of Life & Health Sciences, Aston University, Birmingham B4 7ET, UK; 7 School of BioSciences, The University of Melbourne, Parkville, VIC 3010, Australia; 8 Departamento de Bioquímica, Instituto de Química, Universidade de São Paulo, São Paulo, Brazil

**Keywords:** circadian rhythms, signal transduction, metabolism, sugar signaling

## Abstract

Synchronization of circadian clocks to the day-night cycle ensures the correct timing of biological events. This entrainment process is essential to ensure that the phase of the circadian oscillator is synchronized with daily events within the environment [1], to permit accurate anticipation of environmental changes [2, 3]. Entrainment in plants requires phase changes in the circadian oscillator, through unidentified pathways, which alter circadian oscillator gene expression in response to light, temperature, and sugars [4, 5, 6]. To determine how circadian clocks respond to metabolic rhythms, we investigated the mechanisms by which sugars adjust the circadian phase in *Arabidopsis* [5]. We focused upon metabolic regulation because interactions occur between circadian oscillators and metabolism in several experimental systems [5, 7, 8, 9], but the molecular mechanisms are unidentified. Here, we demonstrate that the transcription factor BASIC LEUCINE ZIPPER63 (bZIP63) regulates the circadian oscillator gene *PSEUDO RESPONSE REGULATOR7* (*PRR7*) to change the circadian phase in response to sugars. We find that SnRK1, a sugar-sensing kinase that regulates bZIP63 activity and circadian period [10, 11, 12, 13, 14] is required for sucrose-induced changes in circadian phase. Furthermore, TREHALOSE-6-PHOSPHATE SYNTHASE1 (TPS1), which synthesizes the signaling sugar trehalose-6-phosphate, is required for circadian phase adjustment in response to sucrose. We demonstrate that daily rhythms of energy availability can entrain the circadian oscillator through the function of bZIP63, TPS1, and the KIN10 subunit of the SnRK1 energy sensor. This identifies a molecular mechanism that adjusts the circadian phase in response to sugars.

## Highlights

• The transcription factor bZIP63 binds and regulates the circadian clock gene *PRR7*
• bZIP63 is required for adjustment of circadian period by sugars
• Trehalose-6-phosphate metabolism and KIN10 signaling regulate circadian period
• Sugar signals establish the correct circadian phase in light and dark cycles

## Graphical Abstract

**Figure:**
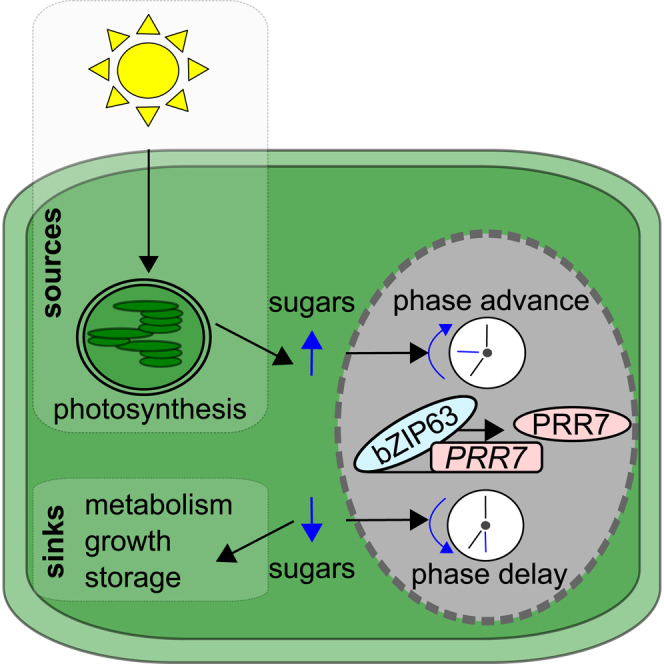

## Teaser

Frank et al. identify mechanisms by which the *Arabidopsis* circadian clock entrains to sugars. Metabolic adjustment of circadian phase involves trehalose-6-phosphate, SnRK1 subunit KIN10 and the transcription factor bZIP63. bZIP63 regulates the circadian clock gene *PSEUDORESPONSE REGULATOR7*. This sets the circadian phase in light and dark cycles.

## Results

### bZIP63 Regulates a Response of the Circadian Oscillator to Sugars

**Figure 1:**
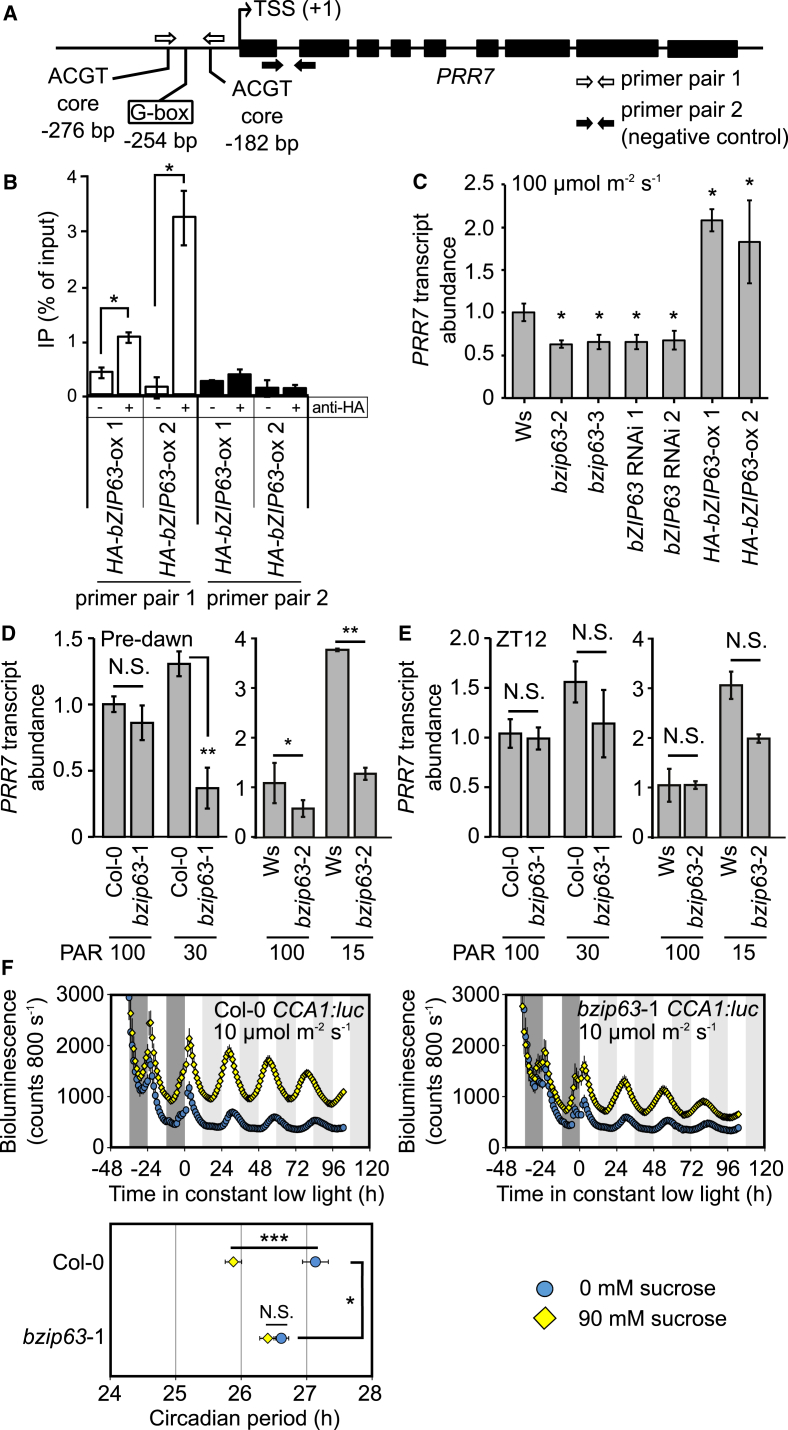
bZIP63 Binds the *PRR7* Promoter to Regulate the Circadian Oscillator (A) *PRR7* structure indicating promoter motifs, transcription start site (TSS), and chromatin immunoprecipitation (ChIP)-PCR primers. Black rectangles indicate exons. (B) bZIP63 binds the *PRR7* promoter (n = 3 (*HA-bZIP63*-ox1) and n = 6 (*HA-bZIP63-ox*2); ±SD); − indicates mock and + indicates immunoprecipitated samples. ChIP used material harvested at end of dark period. (C) *PRR7* transcripts at ZT0 under high light in *bzip63* mutant and RNAi lines, and *bZIP63* overexpressors (n = 3 ± SD; t test). (D and E) bZIP63 regulates *PRR7* transcript abundance in low, but not high, fluence light/dark cycles. *PRR7* transcript abundance immediately before (D) dawn and (E) dusk in mature plants exposed to low light 1 day before sampling (n = 5 ± SD; t test). (C–E) Significance is indicated for comparisons against wild-type at 100 μmol m^–2^ s^–1^. (F) Sucrose shortened the circadian period of *CCA1:luc* in Col-0 (t test), but not *bzip63*-1 (n = 32; ± SEM). Dark and light gray shading indicates actual and subjective darkness, respectively. See also Figure S1.

Circadian entrainment to sugars involves the regulation of *PRR7* transcription [5]. In response to sugars, the wild-type circadian period shortens, whereas that of *prr7*-11 does not [5]. Previous investigation of candidate regulators of *PRR7* failed to identify candidates affecting the response of the circadian oscillator to sugars [5, 15]. We hypothesized that the transcription factor (TF) bZIP63 might regulate *PRR7* because bZIP63 is regulated by the SnRK1 energy sensor and *bZIP63* transcripts peak before *PRR7* in constant light (Figure S1A). bZIP63 is a strong candidate for sugar-mediated regulation of the circadian oscillator because it binds ACGT core element motifs [16], and the *PRR7* promoter contains five ACGT-core bZIP TF-binding motifs within 300 bp of its transcription start site, including a canonical G-box at −254 bp [11, 17, 18, 19] (Figure 1A). bZIP63 binds a *PRR7* promoter region spanning −276 to −182 (Figures 1A and 1B; primer pair 1). *PRR7* transcripts were downregulated in T-DNA insertion mutants and RNAi lines of *bZIP63* (Figures 1C, S1B, and S1C) and upregulated in bZIP63 overexpressors (Figures 1C and S1B). We measured *PRR7* transcript abundance under normal and low light, which mimics starvation, demonstrated by accumulation of the marker transcript *DARK INDUCIBLE6* (*DIN6*) [5, 17] (Figure S1D). Under low-light conditions that deplete endogenous sugars, *PRR7* transcripts accumulated in the wild-type before dawn (Figure 1D [5]), whereas this was attenuated in *bzip63* mutants (Figure 1D). Under high light, which elevates endogenous sugars (Figure S1D [5]), *bzip63* mutations had little effect on *PRR7* transcript abundance; *bzip63*-1 was without effect and *bzip63*-2 reduced *PRR7* transcript abundance slightly (Figure 1D). Sucrose supplementation and high light both suppress *PRR7* transcript accumulation (Figure 1D) [5], and *bzip63* mutants prevent *PRR7* upregulation under low light (Figures 1D and 1E). These data suggest that bZIP63 upregulates *PRR7* in low-energy conditions and that *bzip63*-2 is a stronger allele (Figure 1D). At the end of the photoperiod, the *bzip63* mutations did not affect *PRR7* transcript abundance, consistent with the effects of sugar on PRR7 being restricted to the early photoperiod (Figure 1E) [5]. *PRR7* transcripts decreased in *bzip63*-2 only at the night end, whereas *CIRCADIAN CLOCK ASSOCIATED1* (*CCA1*) and its target *GBS1* were upregulated at ZT20-24 (Figure S1E). Upregulation of *CCA1* in *bzip63*-2 might be due to downregulation of *PRR7*. Therefore, bZIP63 upregulates *PRR7* in response to low energy. This is suppressed by sugars because both sucrose supplementation and *bzip63* mutants inhibit *PRR7* transcript accumulation.

Because we found a *PRR7*-mediated circadian system-wide effect of bZIP63, we investigated whether bZIP63 underlies a response of the circadian oscillator to sugars. Unlike the wild-type, *bzip63*-1 circadian period was unaffected by sucrose under low light (Figure 1F), indicating that the circadian oscillator in *bzip63*-1 is sugar unresponsive. This suggests the circadian oscillator did not respond to sugars in *bzip63*-1 because *PRR7* was not upregulated by low-energy conditions.

### Sugar-Induced Changes in Circadian Period Involve KIN10 and Trehalose-6-Phosphate Biosynthesis

**Figure 2:**
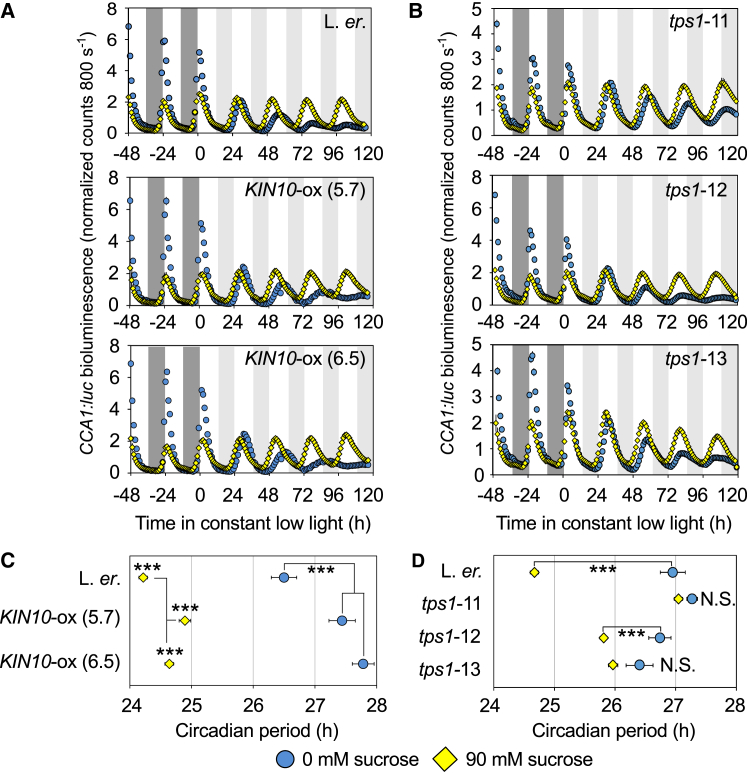
KIN10 and TPS1 Regulate the Response of the *Arabidopsis* Circadian Clock to Sugar (A and B) *CCA1:luc* bioluminescence in low light, with/without exogenous sucrose in (A) two *KIN10-*ox lines (n = 37–58; three experiments combined) and (B) three *tps1* mutants (n = 36–64; six experiments combined). Dark and light gray panels indicate actual and subjective darkness, respectively. (C and D) Circadian period of *CCA1:luc* bioluminescence in KIN10-ox (C) and *tps1* mutants (D), relative to wild-types, with or without exogenous sucrose under low light (t test; ±SEM).

We investigated how regulators of bZIP63 influence the response of the circadian oscillator to sugars. KIN10 (AKIN10/SnRK1.1), an α subunit of the sugar sensor SnRK1 [17], regulates bZIP63 activity in response to starvation [11]. KIN10 overexpression (*KIN10*-ox; Figure S1F) [17] further increased the long circadian period of the wild-type occurring under low-energy conditions (Figures 2A and 2C). The circadian system in *KIN10*-ox remained sugar sensitive because sucrose supplementation shortened its period (Figure 2C) [5]. This could be because overexpressed KIN10 is inhibited post-translationally by sugars. Constitutive KIN10 overexpression under high light, when sugars are high, did not lengthen the period (Figure S2A).

Under low-energy conditions, such as low light, KIN10-ox caused a longer period relative to the wild-type (Figure 2C). Under high-energy conditions (either low light plus sucrose or high light), KIN10-ox had less effect (Figures 2C and S2A). Therefore, sugar levels affect the circadian phenotype in KIN10-ox. This is consistent with KIN10 regulating the circadian clock in response to energy status because low-energy conditions cause a longer period [5], KIN10 is activated by low energy [14, 17], and sucrose rescues light-intensity-dependent effects of KIN10 upon the circadian oscillator [14].

TREHALOSE-6-PHOSPHATE SYNTHASE1 (TPS1) synthesizes the signaling sugar trehalose-6-phosphate (Tre6P). Tre6P concentration tracks sucrose and negatively regulates SnRK1 activity, bZIP63, and other sugar-sensing targets [11, 20, 21]. We investigated the circadian phenotype of three hypomorphic *tps1* TILLING mutants [22]. Sucrose had no effect on period in *tps1-*11 and *tps1-*13 (Figures 2B and 2D). Sucrose shortened the period of *tps1-*12, the weakest allele for metabolite alterations [22], but less than the wild-type (Figure 2D). In the presence of sucrose, all three *tps1* alleles had longer periods than the wild-type (Figure 2D), presumably because low Tre6P mimics starvation. In the absence of sucrose under low-light conditions, *tps1* mutants did not have a longer period than the wild-type (Figure 2D). This might be because these seedlings were already in a low-sugar state [5], so disrupting this pathway caused no further change.

### Diel and Circadian Rhythms of Sugar Signaling Revealed by DIN6 Promoter Dynamics

**Figure 3:**
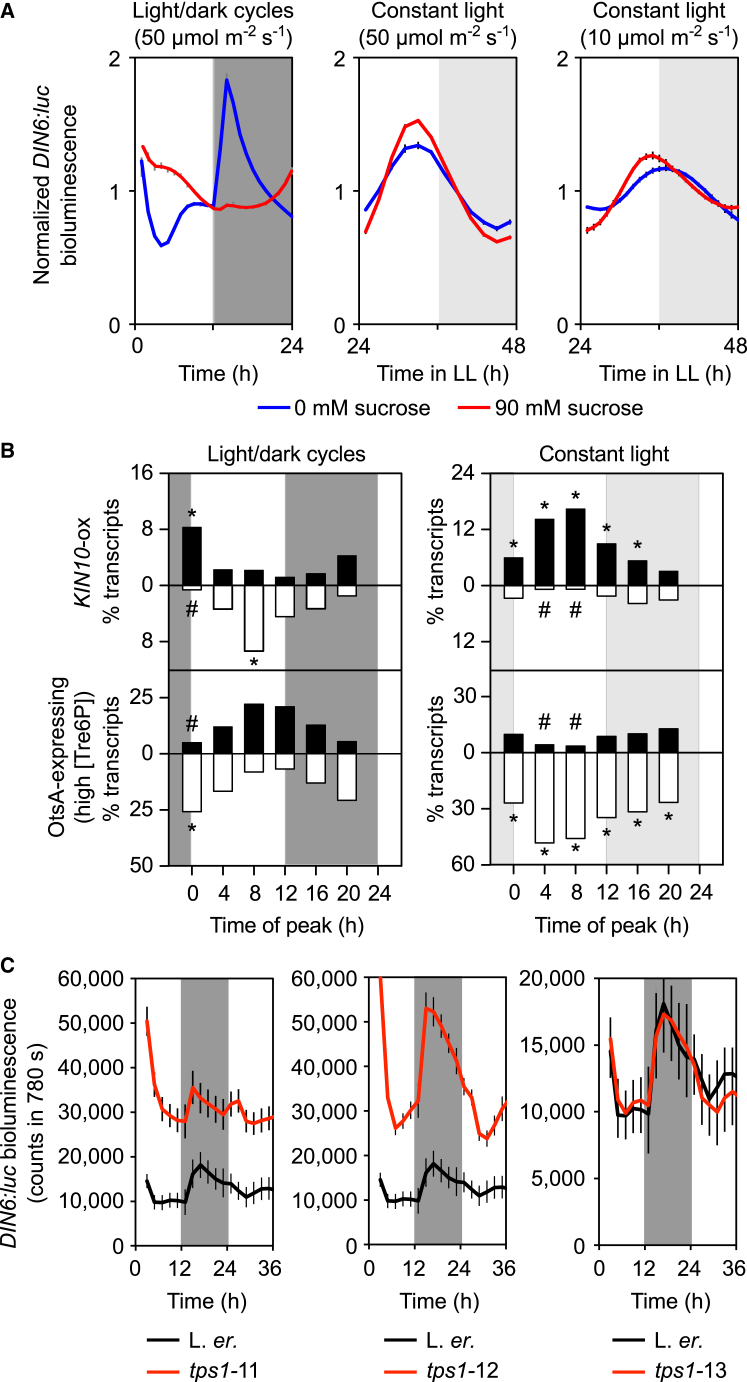
Diel Cellular Energy Signaling Dynamics (A) Normalized *DIN6:luc* bioluminescence dynamics (n = 6; ±SEM). (B) A significant proportion of circadian [24, 25, 26] and diel-regulated [23, 27] transcripts are regulated by KIN10 signaling [17, 20]. This analysis combines individual datasets from Figures S2E and S2F. Transcripts binned by phase (upregulated, black; downregulated, white). ^∗^ and # indicate overlaps with more or fewer transcripts than expected from a chance association between gene sets, respectively. (C) Under light/dark, *DIN6:luc* is upregulated in *tps1*-11 and *tps1*-12 (n = 6; ±SEM). Dark and light gray shading indicates actual and subjective darkness, respectively. See also Figures S2 and S3.

Energy changes might arise from fluctuations in sucrose availability [23]. We investigated this using *DARK INDUCIBLE6* (*DIN6*/*ASN1*), which is upregulated by starvation, downregulated by sugars, and downstream of the regulation of bZIP63 by KIN10 [11, 17]. *DIN6:luc* had a diel rhythm, with promoter activity (2.4-fold; Figure 3A) and transcript abundance (2.7-fold; Figure S2B) increasing after dusk. *DIN6* promoter activity reduced through the night, presumably as sugars became available from starch breakdown [28]. After dawn, *DIN6:luc* activity decreased rapidly, suggesting the accumulation of photosynthetic sugars (Figure 3A). *DIN6* promoter dynamics in light/dark cycles arose from sugar status alterations, because sucrose supplementation attenuated *DIN6:luc* rhythms (Figure 3A). This is also supported by increased *DIN6* transcript abundance under low-light/dark cycles at pre-dawn and dusk compared with high-light controls (Figure S1D). *DIN6* promoter activity and transcript accumulation are also circadian regulated, peaking in the middle of the subjective day in constant high light (LL) (Figures 3A and S2C). As with *CCA1:luc* [5], *DIN6:luc* was phase-advanced by sucrose under constant low light but not higher light intensity. Therefore, under light/dark cycles there are sugar-dependent cycles in a readout of KIN10- and bZIP63-mediated sugar signaling [11, 17]. This indicates that starvation pathways are upregulated during each night of light-dark cycles (Figures 3A and S2B). Extensive integration of circadian regulation with energy signaling is corroborated by statistically significant overlaps between KIN10- and [Tre6P]-regulated transcripts and five sets of circadian- and diel-regulated transcripts (Figures 3B and S2D–S2F) [17, 23, 24, 25, 26, 27].

Diel cycles of *DIN6:luc* activity are likely mediated by Tre6P. *DIN6:luc* activity was increased in two *tps1* alleles (*tps1-*11 and *tps1-*12), particularly at night when low sugar availability combines with the low TPS1 activity in these mutants to activate KIN10 and the *DIN6* promoter [11, 17, 20] (Figure 3C). *DIN6:luc* was unaltered in *tps1*-13, which is a weaker allele for some physiological traits [22]. This demonstrates that Tre6P transmits diel changes in sugar status to the bZIP63-responsive promoter *DIN6* (Figure 3C) [17, 18].

### Circadian Oscillator Protein CHE Interacts with bZIP63 and Regulates a Response of the Circadian Oscillator to Sugar

bZIP TFs undergo regulatory interactions with many other proteins [29]. We investigated whether these might contribute to circadian entrainment by sugars. Using a yeast two-hybrid (Y2H) screen, we identified interaction between bZIP63 and the circadian oscillator component CCA1 HIKING EXPEDITION (CHE/TCP21) (Figure S3A). Like PRR7, CHE is a transcriptional repressor of *CCA1* [30, 31]. No other known regulators of the circadian system interacted with bZIP63. We confirmed that bZIP63 interacts with CHE *in planta* using bimolecular fluorescence complementation (Figure S3B). We hypothesized that CHE might contribute to a response of the circadian oscillator to sugars. We found that sucrose induces *CHE* transcripts in the wild-type, but this was attenuated in *KIN10*-ox and somewhat in *tps1-*12 (Figure S3C). In light/dark cycles without sucrose supplementation, *CHE* overexpression and *che* loss-of-function mutants suppressed and increased the amplitude of daily *CCA1:luc* fluctuations, respectively (Figures S3D and S3E) [30]. When diel changes in sugar status were eliminated by cultivation on 90 mM sucrose (Figure 3A), the amplitude difference between *che* mutants and the wild-type was abolished (Figures S3D and S3E). This suggests that CHE might not suppress *CCA1* under sugar-replete conditions, and that CHE regulates a response of *CCA1* to sugars. This occurred independently from CHE binding to the TCP-binding site (TBS) within the *CCA1* promoter [30], because mutation of the TBS did not alter the response of *CCA1* to a morning sugar pulse (Figure S3F).

### bZIP63, KIN10, and TPS1 Regulate the Response of Circadian Phase to Sugars

**Figure 4:**
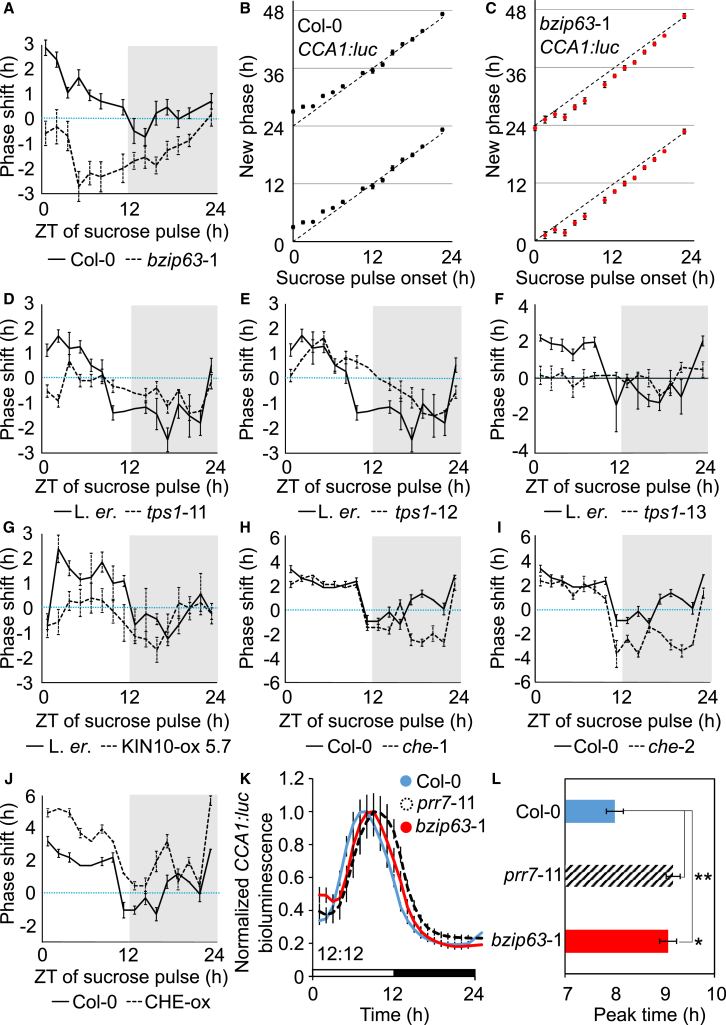
TPS1, KIN10, and bZIP63 Entrain the Circadian Oscillator (A–J) Phase response curves (A and D–J) and phase transition curves (B and C) of *CCA1:luc* for sucrose treatment of *bzip63*-1 (A), *tps1*-11 (D), *tps1*-12 (E), *tps1*-13 (F), KIN10-ox (G), *che*-1 (H), *che*-2 (I), and *CHE*-ox (J). x axes indicate zeitgeber time (ZT) of sugar pulse. Blue dotted line indicates phase of a control grown without sucrose. (K and L) Phase of rhythms of *CCA1:luc* in Col-0, *prr7-*11 and *bzip63-*1 in light/dark cycles of 70 μmol m^–2^ s^–1^ in the absence of sucrose (n = 12 ± SEM; t test), plotted as *CCA1:luc* bioluminescence (K) and time of peak bioluminescence (L). Shaded areas indicate subjective dark period. In (B) and (C), phase transition curves are double-plotted using data from (A) and indicate new phase against time following a 90 mM sucrose pulse for (B) wild-type and (C) *bzip63*-1. Dashed line indicates no phase shift. Data from two independent experiments were combined (n = 8 in each; ±SEM).

To test the potential involvement of bZIP63, KIN10, Tre6P, and CHE in circadian entrainment, we measured the time-dependent adjustment of circadian phase in response to a sucrose pulse. This tests the response of the circadian oscillator to a transient stimulus, as opposed to prolonged sucrose treatments (Figures 1F and 2). A morning sugar pulse (ZT0–ZT6) advanced the wild-type circadian phase (Figures 4A and 4B) [5]. In contrast, morning sugar pulses did not advance the circadian phase in *bzip63*-1 mutants (Figures 4A and 4C). This demonstrates that sucrose acts as a type 1 (weak) zeitgeber in the wild-type, resulting in a circadian phase advance. This phase advance was absent in *bzip63*-1. There was also no phase advance in *tps1*-11, *tps1*-13, and *KIN10*-ox in response to morning sucrose pulses (ZT3–ZT7.5, Figures 4D–4G). *tps1*-12 had a phase advance in response to sucrose pulses at this time (Figure 4E), which is consistent with its weaker metabolic phenotypes [22]. These data suggest that TPS1, KIN10, and bZIP63 might be positioned within a pathway by which sugars entrain the circadian oscillator (Figures 4A–4G).

Two *che* mutations had little effect upon the morning sucrose-induced phase advance (ZT0–ZT7.5; Figures 4H and 4I), and a sucrose-induced phase delay occurred in *che* during the subjective night (ZT15–ZT19.5; Figures 4H and 4I). *CHE*-ox increased the magnitude of the sucrose-induced phase advance of *CCA1* at most times of day (Figure 4J). Therefore, CHE is not required for the sugar-induced circadian phase advance in the morning but might be associated with other sugar responses. This is consistent with our hypothesis that circadian entrainment to sugars depends upon *PRR7*, with the alteration of *CCA1* transcription occurring in response *PRR7* expression dynamics [5].

Lastly, we examined whether PRR7 and bZIP63 are required for correct circadian function under day/night cycles. *prr7-*11 and *bzip63-*1 have a late phase of *CCA1* expression (Figures 4K and 4L) demonstrating that bZIP63 and PRR7 are required for correct oscillator phase under light/dark cycles (Figures 4K and 4L). These late-phase phenotypes (Figures 4K and 4L) suggest defective entrainment. Considering that bZIP63 participates in the regulation of the circadian oscillator in response to sugars (Figure 1F), this sugar-responsive entrainment pathway is required to ensure correct circadian phase under light/dark cycles.

## Discussion

Our finding that bZIP63 regulates the circadian oscillator allowed us to investigate entrainment of the *Arabidopsis* circadian oscillator by sugars. We propose that daily fluctuations in sugar availability might be signaled by Tre6P to effect entrainment. Mutants impaired in Tre6P production had a reduced response of circadian period to sucrose (Figures 2B and 2D), and the circadian oscillator of *tps1* mutants was not entrained by sucrose (Figures 4D–4F). bZIP63 homo- and heterodimerization is regulated by KIN10-mediated phosphorylation [11], so transcriptional regulation of *PRR7* by bZIP63 might arise from bZIP63 phosphorylation dynamics. Mutants of both *bzip63* and its negative regulator TPS1 have similar effects on the circadian oscillator because *PRR7* cannot respond to sugar dynamics in both sets of mutants. *KIN10*-ox was insensitive to entraining sugar pulses, suggesting that SnRK1 participates in entrainment to transient sugar fluctuations (Figure 4G). Morning sucrose pulses delayed the phase in *bzip63*-1 (Figure 4A), whereas the phase in *tps1*-11 and *tps1*-13 was sucrose insensitive (Figures 4D and 4F). This difference might reflect additional effects of the *tps1* TILLING alleles, which influence a range of phenotypes [22]. Other kinases and pathways might be involved because *KIN10*-ox seedlings retained a shorter period, like the wild-type, during long-term sucrose supplementation (Figure 2C) [14]. *bzip63-*1 was unresponsive to prolonged sucrose stimulation (Figure 1F), whereas it retained some sucrose-induced phase changes within the phase response curve (Figure 4A). This is similar to the *lip1*-1 mutant, which has a very small period response to varying intensity of continuous light, but retains a phase delay response to pulsed light in phase response curves [32]. In continuous darkness, the period of an evening reporter of the circadian oscillator (*GIGANTEA*) is unaltered in KIN10-ox [14]. This might be because, in continuous darkness, evening components of the circadian oscillator appear to uncouple from morning components such as *CCA1* and *PRR7* investigated here [5, 15, 33].

We found that bZIP63 binds the *PRR7* promoter in a region containing a canonical G-box motif. While bZIP63 might bind to other *cis* elements within this region, for two reasons it is possible that bZIP63 binds to this −254 bp G-box. First, mutating the G-box within the promoter of another bZIP63- and KIN10-regulated gene, *DIN6*, abolishes the regulation of *DIN6* by KIN10 [17]. Second, bZIP1 binding to G-box motifs is enhanced by heterodimerization with bZIP63 [34, 35].

Our data suggest that the response of bZIP63 and PRR7 to sugar entrains the *Arabidopsis* circadian clock to dynamic energy signals during light/dark cycles. This will allow identification of other network components, since bZIP63 and other bZIPs are phosphorylated by protein kinases other than KIN10, potentially including KIN11 and casein kinase II [11, 29]. It will be informative to determine whether other bZIP TFs dimerize with bZIP63 to regulate *PRR7* transcription and whether binding and/or activity of bZIP63 is regulated by energy status.

bZIP63 works through protein-protein interactions in addition to protein-DNA interactions. We identified potential interactions with CHE, a regulator of *CCA1* [30]. This suggests several modes of regulation of bZIP63 activity and that CHE might affect the circadian oscillator through multiple mechanisms. bZIP63 interacts with a further TCP TF, TCP2 [36], suggesting an interacting network of bZIP and TCP TFs. This bZIP63-CHE interaction provides further evidence that TCP TFs are important regulators within the plant circadian system [30, 37, 38, 39]. The bZIP63-CHE interaction might have mechanistic similarities with the coincident binding of interacting TOC1 and PIF3 to promoters of growth-regulating genes [40]. PIF4 is proposed as an additional regulator of circadian entrainment to sugars [41], although PIF4 does not bind the *PRR7* promoter [42, 43] and roles for PIF4 within metabolic entrainment remain untested.

### Conclusions

Our identification of key molecular components that permit the circadian oscillator to respond to sugars brings a new dimension to the study of plant circadian systems, by identifying a TF that adjusts circadian phase to entrain the oscillator. Our finding that bZIP63 upregulates *PRR7* promoter activity in response to low energy suggests that sugars regulate circadian period and phase through a signaling pathway rather than indirect metabolic changes [44]. This underlies the plasticity of the circadian period to sugars, with this plasticity absent from *prr7-*11 and *bzip63* mutants (Figure 1F) [5]. We propose that the dynamic sensitivity of the circadian system to cellular energy, through bZIP63 regulation of *PRR7* expression, permits its continuous metabolic adjustment to contribute to energy homeostasis [45]. This is important because circadian systems provide a selective advantage through their phase relationship with the environment [3, 46]. Here, we identified a mechanism that establishes that phase relationship.

## STAR★Methods

### Key Resources Table

**Table:** 

REAGENT or RESOURCE	SOURCE	IDENTIFIER
**Antibodies**		

KIN10 antibody	Agrisera	Cat# AS10 919; RRID: AB_10754154
RbcL antibody	Agrisera	Cat# AS03 037A; RRID: AB_2175408
Anti-HA antibody	Santa Cruz Biotechnology	Cat# sc-7392 C1313
H3-K9 antibody	Epigentek	Cat# P-2014-48
Normal mouse IgG antibody	Epigentek	Cat# P-2014-48

**Bacterial and Virus Strains**		

*Escherichia coli* DH5α	Thermo-Fisher	Cat# 18265017
*Escherichia coli* DB3.1	Thermo-Fisher	Cat# A10460
*Agrobacterium tumefaciens* C58C1	N/A	N/A
*Agrobacterium tumefaciens* GV3101	N/A	N/A

**Chemicals, Peptides, and Recombinant Proteins**		

Bacto-agar	VWR	Cat# 214050
Duchefa Murashige & Skoog Medium	Melford Laboratories	Cat# M0221.0050
Sucrose	Thermo-Fisher	Cat# 10020440
Sorbitol	Thermo-Fisher	Cat# BP439-500
3′,5′-dimethoxy-4’-hydroxyacetophenone (Acetoseringone)	Sigma-Aldrich	Cat# D134406
Kanamycin	GIBCO	Cat# 11815-024
Ampicillin	Sigma-Aldrich	Cat# A0166
Rifampicin	Affymetrix	Cat# USB-21246
Tetracycline	Affymetrix	Cat# USB-22105
Phosphinothricin	Melford Laboratories	Cat# P0159.0250
BamHI	New England Biolabs	Cat# R0136S
KpnI	New England Biolabs	Cat# R0142S
T4 DNA ligase	New England Biolabs	Cat# M0202S
SD/-His/-Leu/-Trp/-Ura with Agar	Clontech	Cat# 630325
Drop-out Supplement -Leu/-Trp	Clontech	Cat# 630417
Drop-out Supplement -His/-Leu/-Trp	Clontech	Cat# 630419
3-amino-1,2,4-triazole (3AT)	Sigma-Aldrich	Cat# A8056
Sterile RNase-free water	Thermo-Fisher	Cat# BP561-1
RNaseZap RNase decontamination solution	Thermo-Fisher	Cat# AM9780
Phusion High-Fidelity DNA Polymerase	New England Biolabs	Cat# M0530S
D-luciferin, potassium salt	Melford Laboratories	Cat# L37060

**Critical Commercial Assays**		

EpiQuik Plant ChIP kit	Epigentek	Cat# P-0214-048
pGEM-T easy vector systems kit	Promega	Cat# A1360
pENTR/D-TOPO cloning kit, with One Shot TOP10 chemically competent *E. coli*	Thermo-Fisher	Cat# K240020
ProQuest two-hybrid system with Gateway Technology	Thermo-Fisher	Cat# PQ1000101
RNEasy Plant Mini kit	QIAGEN	Cat# 74104
Machery-Nagel Nucleospin II RNA kit	Thermo-Fisher	Cat# 12373368
Machery-Nagel Nucleospin Plasmid kit	Thermo-Fisher	Cat# 11932392
High-Capacity cDNA reverse transcription kit	Life Technologies	Cat# 4368814
RNAase inhibitor for reverse transcription kit	Life Technologies	Cat# N8080119
Brilliant III Ultra-Fast SYBR Green QPCR Master Mix	Agilent Technologies	Cat# 600883

**Experimental Models: Cell Lines**		

*Saccharomyces cerevisiae* strain PJ694a (MATa, trp1-901, leu2-3,122, ura3-52, his3-200 gal4Δ, gal80Δ, LYS2::GAL1-HIS3, GAL2-ADE2::GAL7-lacZ)	[47]	N/A

**Experimental Models: Organisms/Strains**		

*Arabidopsis*: Col-0	Nottingham *Arabidopsis* Stock Centre	N/A
*Arabidopsis*: Wassilewskija	Nottingham *Arabidopsis* Stock Centre	N/A
*Arabidopsis*: Landsberg *erecta*	Nottingham *Arabidopsis* Stock Centre	N/A
*Arabidopsis*: *bzip63*-1	[16]	N/A
*Arabidopsis*: *bzip63*-2	[16]	N/A
*Arabidopsis*: *bzip63*-3	Nottingham *Arabidopsis* Stock Centre	Line FLAG_532A10
*Arabidopsis*: bZIP63 RNAi 1	This paper	N/A
*Arabidopsis*: bZIP63 RNAi 2	This paper	N/A
*Arabidopsis*: HA-bZIP63-ox 1	This paper	N/A
*Arabidopsis*: HA-bZIP63-ox 2	This paper	N/A
*Arabidopsis*: KIN10-ox 5.7	[15]	N/A
*Arabidopsis*: KIN10-ox 6.5	[15]	N/A
*Arabidopsis*: *tps1*-11	[22]	N/A
*Arabidopsis*: *tps1*-12	[22]	N/A
*Arabidopsis*: *tps1*-13	[22]	N/A
*Arabidopsis*: *prr7*-11	[48]	N/A
*Arabidopsis*: *che*-1	[30]	N/A
*Arabidopsis*: *che*-2	[30]	N/A
*Arabidopsis*: CHE-ox	[30]	N/A
*Nicotiana benthemiana*	N/A	N/A

**Oligonucleotides**		

See Table S1	N/A	N/A

**Recombinant DNA**		

pGREENII 0229 binary vector	John Innes Centre, U.K.	pGREENII0229
pSOUP helper vector	John Innes Centre, U.K.	pSOUP
CCA1:luc binary vector	[49]	N/A
pPZP CCA1(TBSm):luc	[30]	N/A
pPZP CCA1:luc	[30]	N/A
pDEST22	Thermo-Fisher	Cat# PQ1000101
pDEST32	Thermo-Fisher	Cat# PQ1000101
pDEST32:CHE	This paper	N/A
pDEST32:bZIP63	This paper	N/A
pDEST22:bZIP63	This paper	N/A
HA-bZIP63-ox in pFP101HAVP16	[50]	N/A
bZIP63 RNAi in pHANNIBAL	[51]	N/A
bZIP63 RNAi in pFP100-LacZ	[50]	N/A
pSPYNE-35S (YFPN)	[52]	N/A
pSPYCE-35S (YFPC)	[52]	N/A
pSPYNE-35S:bZIP63 (bZIP63-YFPN)	This paper	N/A
pSPYCE-35S:bZIP63 (bZIP63-YFPC)	This paper	N/A
pSPYCE-35S:CHE (CHE-YFPC)	This paper	N/A
pCH32	[53]	N/A

**Software and Algorithms**		

Excel	Microsoft	N/A
Sigmaplot 13.0	Systat Software, USA	N/A
Inkscape 0.91	https://inkscape.org/en/	N/A
Biological Rhythms Analysis Software System (BRASS)	University of Edinburgh; http://millar.bio.ed.ac.uk/	N/A
Image32	Photek, U.K.	N/A

**Other**		

MLR350/352 growth chamber	Sanyo or Panasonic, Japan	N/A
Photek HRPCS intensified CCD camera system	Photek, U.K.	N/A
LB982 Nightshade	Berthold Technologies, Germany	N/A
GFP2 filter equipped SMZ1000 stereomicroscope	Nikon	N/A
LSM 510 Confocal Microscope	Zeiss	N/A
Zen software	Zeiss	N/A
Mx3005P real-time PCR machine	Agilent Technologies	N/A

### Contact for Reagent and Resource Sharing

Further information and requests for resources and reagents should be directed to and will be fulfilled by Antony Dodd (antony.dodd@bristol.ac.uk).

### Experimental Model and Subject Details

*Arabidopsis thaliana* (L.) Heynh. background lines Columbia-0 (Col-0), Landsberg *erecta* (L. *er*.) and Wassilewskija (Ws) were used for experimentation, with mutants and transgenic lines in these backgrounds as detailed in the Key Resources Table. *Arabidopsis* seedlings were cultivated at 19°C, under light conditions required by each experiment and described in the Results. *Nicotiana benthemiana* was cultivated at 25°C (both for growth and bimolecular fluorescence complementation analysis). *Saccharomyces cerevisiae* was cultured at 30°C for all assays.

### Method Details

#### Plant material and growth conditions

Seeds were surface-sterilized with 10% v/v sodium hypochlorite (Fisher Scientific, Loughborough, UK) and 0.02% (v/v) Triton X-100 (Fisher Scientific, Loughborough, UK) for 5 min, washed three times with sterile deionised water and sown on half-strength Murashige & Skoog media (Duchefa, Netherlands), pH = 5.7 with 0.8% w/v agar (Bactoagar, BD). Where specified, media was supplemented with 90 mM sucrose or 90 mM sorbitol as an osmotic control. This concentration of sucrose is appropriate for our experiments because it saturates the sugar response of the circadian oscillator, is the standard concentration of sucrose used for experimentation with *Arabidopsis*, and there is no dose-dependent effect of sucrose upon circadian entrainment [5, 14, 15, 25, 33]. Seeds were stratified at 4°C for 2 or 3 days in darkness, then transferred into 50 μmol m^-2^ s^-1^ (before starting low light experiments) or 80 – 100 μmol m^-2^ s^-1^ (for standard light experiments) photon flux of cool white fluorescent light, at 19°C, with cycles of 12 hr light and darkness (MLR-350/352 growth chamber, Sanyo/Panasonic, Japan). Background lines for the *tps* mutants were derived originally from mutagenesis of Col-0 and backcrossed three times with Landsberg *erecta* (L. *er.*) [22]. We have, therefore, used L. *er.* as a control for experiments with *tps* lines. KIN10-ox was as described elsewhere [17]. T-DNA insertion mutants *bzip63-*1 (SALK_006531, Col-0 background [18]), *bzip63-*2 (FLAG_610A08, Ws-2 background [18]) and *bzip63-*3 (FLAG_532A10, Ws-2 background) were obtained from the *Arabidopsis* Biological Resource Center (ABRC). Homozygous *bzip63-3* was isolated using kanamycin-resistance segregation analysis, and sequencing of the flanking regions revealed that the T-DNA is inserted in the sixth exon of the *bZIP63* gene. For experiments using mature plants, *Arabidopsis* was grown under 12 hr photoperiods of 100 μmol m^-2^ s^-1^ for 30 days before experimentation.

#### Generation of transgenic lines

To make *HA-bZIP63*-ox and *bZIP63* RNAi lines, both the *bZIP63* coding sequence (CDS) and a 350 bp fragment for RNAi were amplified using PCR primers that incorporated restriction sites (indicated in lower case); overexpressor: tctaga ATGGAAAAAGTTTTCTCC (FP); ggatccCTACTGATCCCCAACGCT (RP); RNAi: aagcttggtaccTCACTGGTCGGTTAATGG (FP); tctagactcgagCACTTGTTATAGCACTGC (RP). The *bZIP63* CDS was cloned into the pFP101 vector, fused to 3xHA tag and the VP16 activation domain for overexpression by the CaMV 35S promoter. The 350 bp *bZIP63* fragment was cloned antisense and sense into pHANNIBAL, then transferred to pFP100-Lacz with the CaMV 35S promoter driving antisense-sense hairpin expression.

To produce the *DIN6:luciferase* construct, 2659 bp of genomic DNA upstream of the *DIN6* start codon was isolated by PCR, using primers that introduced the KpnI (5′) and HindIII (3′) restriction sites (FP: CGTGGTACCTGGACATGAGTGCATGAC; RP: GCGAAGCTTGAAGAAAGTGAAAAAGATCACG). The fragment was ligated into a modified pGreenII0179 binary vector containing the *LUCIFERASE+* coding sequence [54]. The *CCA1:luciferase* construct used is described elsewhere [55]. Reporter lines with *CCA1(TBSm):luc* and control *CCA1:luc* used constructs in the pPZP binary vector described elsewhere [30]. All constructs were transformed into *Arabidopsis* by the floral dip method, using *Agrobacterium tumefaciens* strain GV3101. Transformants were identified with hygromycin (pGreenII0179) or gentamycin (pPZP) selection, or from GFP fluorescence in the seed coat (pFP101 and pFP100-LacZ), using a GFP2-equipped SMZ1000 stereomicroscope. Lines were validated by PCR and RT-PCR, and selected for similar levels of luciferase activity. Homozygous third or fourth generation seed lines were used for experiments.

#### Bioluminescence imaging

Imaging of luciferase bioluminescence was performed as described previously [5, 54]. Briefly, circular clusters of 9 day old seedlings were supplied with 2 mM (two doses) or 5 mM (single dose) of D-luciferin potassium salt (Melford Labs, UK, or Biosynth AG, Switzerland) between 1 hr and 24 hr prior to commencing imaging. Luciferase bioluminescence was integrated for 800 s each hour using either a Photek HRPCS intensified CCD camera (Photek, Hastings, UK) or LB 982 NightSHADE (Berthold Technologies, Bad Wildbad, Germany). Light was controlled automatically to provide the stated photon irradiance and LD cycles or constant (LL) light from a red/blue LED mix (wavelengths 660 nm and 470 nm). Circadian oscillation parameters were calculated from four 24 hr cycles, excluding the first 24 hr of data, using the Fast Fourier Transform Non-Linear Least-squares method [56] within the BRASS software (http://millar.bio.ed.ac.uk/). The mean peak height parameter was calculated as an average of the differences between peak (maximum value) and trough (minimal value) measured on days 2, 3, 4, 5 and 6 of LL (thus excluding first 24 hr), and for LD was calculated by averaging the differences between peaks and troughs measured for each day under light/dark conditions. Phase response curves were produced using the same method as described previously [5, 57].

#### RNA extraction and real time PCR analysis

Sampling and RNA isolation for real-time PCR was performed as described previously [5, 54]. Primers were *PRR7*: TTCCGAAAGAAGGTACGATAC (FP); GCTATCCTCAATGTTTTTTATGT (RP); *PP2AA3* reference transcript [18]: CATGTTCCAAACTCTTACCTG (FP); GTTCTCCACAACCGCTTGGT (RP) (for Figure 1C); *CHE*: TAATGGGTGGTGGTGGTTCTG (FP); GCAAAGCTCCAGACTTGTCC (RP); Figure S3C); *DIN6*: TTCACCTTTCGGCCTACGAT (FP); ATCGGCATGTTGTCAATTGC (RP); *ACT2* reference (TGAGAGATTCAGATGCCCAGAA (FP); TGGATTCCAGCAGCTTCCAT (RP) (for Figure S3C).

#### Chromatin immunoprecipitation

Aerial parts of 12-day old seedlings were vacuum-infiltrated with 1% v/v formaldehyde solution and incubated at room temperature for 20 min to crosslink DNA-protein complexes. After cross-linking, tissues were ground in liquid nitrogen using mortar and pestle. Chromatin extraction was performed as described elsewhere [58]. The isolated chromatin was re-suspended in 300 μL of nuclear lysis buffer (50 mM Tris-HCL, pH 8; 10 mM EDTA; 1% w/v SDS; 1X Pierce protease inhibitor cocktail #88265 (Thermo Scientific, Massachusetts, USA). Resuspended chromatin was sonicated to achieve DNA fragments ranging 0.3 - 1 Kb. Immunoprecipitation was performed using EpiQuik Plant ChIP kit (Epigentek Group, Farmingdale, USA) following manufacturer’s instructions. DNA-protein complexes were immuno-precipitated using a monoclonal anti-HA antibody (Santa Cruz Biotechnology, Texas, USA). Analysis of enrichment of target genes was performed by qPCR (ABI 7500 Fast Real-time PCR System). For *PRR7*, primer pair 1 was GACGTTTTCCTTACCCACCA (FP), ATTGGCGAGGATTAGTGACG (RP), and primer pair 2 TGCTTTTGTATGGTTGGATTTTT (FP), TGAAGAACGACGAATTCTCAAA (RP) (Figures 1A and 1B). Data were normalized using cycle thresholds from immunoprecipitated and non-immunoprecipitated samples as elsewhere.

Chromatin immunoprecipitation (ChIP)-qPCR was performed using two transgenic lines overexpressing HA-tagged bZIP63 (*HA-bZIP63*-ox1 and *HA-bZIP63-ox*2 in Figure 1B), using an anti-HA antibody (+) and an anti-mouse IgG antibody (-) as a negative control. ChIP using overexpressor lines is a common approach (e.g., [59, 60]), with the bZIP63 overexpressors accumulating approximately double the transcript as the wild-type under high light at ZT0 (Figure 1C). Primer pair 1 amplifies the promoter region containing G-box motif and primer pair 2 as a control amplifies a region without any putative bZIP binding site. Data in Figure 1B compare the cycle threshold (Ct) of the anti-HA antibody IP samples (adjusted relative to Ct of input DNA) and Ct of mock IP (adjusted relative to Ct of the input DNA); t tests compared control and anti-HA treated samples (*HA-bZIP63*-ox1 p = 0.011; *HA-bZIP63*-ox2 p = 0.014).

#### Bimolecular fluorescence complementation

Full-length coding regions of *bZIP63* (AT5G28770.2) and *CHE* (AT5G08330.1) were cloned using specific primers (CHE: TAAGCAGGATCCATGGCCGACAACGACGGAGC (FP); TAAGCAGGTACCACGTGGTTCGTGGTCGTC (RP); bZIP63: TAAGCAGGATCCATGGAAAAAGTTTTCTCCG (FP); TAAGCAGGTACCCTGATCCCCAACGCTTC (RP) into binary vectors pSPYNE-35S (YFP^N terminus^; aa 1-155) or pSPYCE-35S (YFP^C terminus^; aa 156-239) to generate the fusion proteins bZIP63-YFP^N^, bZIP63-YFP^C^ and CHE-YFP^C^ [52]. The binary vectors containing the constructs bZIP63-YFP^N^, bZIP63-YFP^C^ and CHE-YFP^C^ were inserted into *Agrobacterium tumefaciens* strain C58C1, which was grown in Luria-Bertani (LB) liquid medium containing appropriate antibiotics (100 μg ml^-1^ rifampicin, 50 μg ml^-1^ kanamycin, 10 μg ml^-1^ tetracycline) at 28°C for 16 hr and 200 RPM agitation. Bacterial cells were harvested by centrifugation and the pellet resuspended in 5 mL of infiltration buffer (10 mM MgCl_2_, 10 mM MES-KOH pH 5.7 and 200 μM acetosyringone (#D134406, Sigma-Aldrich). *Agrobacterium* strains carrying the constructs containing putative interacting proteins were mixed and co-infiltrated in the abaxial surface of four-week-old *Nicotiana benthamiana* leaves at a final OD_600_ = 0.5 each. To enhance expression of fusion proteins, *Agrobacterium* C58C1 carrying the pCH32 helper plasmid that suppress gene silencing [53] was co-infiltrated in all experiments. After 3-4 days, infiltrated regions of *Nicotiana* leaves were excised and pavement cells visualized in a confocal laser scanning microscope (LSM 510 Meta, Carl Zeiss, Thornwood, NY, U.S.A.) with an argon laser (excitation = 488 nm, emission = 524 nm).

#### Yeast two-hybrid analysis

Full-length coding regions of *bZIP63* (AT5G28770.2) and *CHE* (AT5G08330.1) were cloned using specific primers (*CHE*: caccATGGCCGACAACGACGGAGC (FP); TCAACGTGGTTCGTGGTCGTC (RP); *bZIP63*: caccATGGAAAAAGTTTTCTCCGAC (FP); CTACTGATCCCCAACGCTTC (RP) into pENTR-D-TOPO (Thermo Scientific) following the manufacturer’s instructions, to generate pENTR-D-TOPO:bZIP63 and pENTR-D-TOPO:CHE intermediary entry vectors. The sequence indicated in lowercase in the *bZIP63* and *CHE* forward primers (FP) were incorporated by PCR into the amplicon for directional cloning in pENTR-D-TOPO. Subsequently, the intermediary entry vectors were recombined into the yeast expression vectors pDEST32 and pDEST22 of the ProQuest Two-Hybrid System with Gateway Technology (Thermo Scientific) following manufacturer’s instructions. The resulting yeast expression vectors pDEST32:bZIP63; pDEST32:CHE; and pDEST22:bZIP63, as well as the empty vectors pDEST32 and pDEST22 (used here as negative controls) were transformed into *Saccharomyces cerevisiae* strain PJ69-4a (*MATa trp1-901 leu2-3,122 ura3-52 his3-200 gal4Δ gal80Δ LYS2::GAL1-HIS3 GAL2-ADE2::GAL7-lacZ*) [47]. Y2H assays were performed using yeast double-transformants (pDEST32:bait + pDEST22:prey) lines. The yeast lines obtained were grown for 16 hr (overnight) at 30°C and 200 RPM agitation in minimal SD (Synthetic Defined) liquid medium lacking leucine and tryptophan (SD/-Leu/-Trp), auxotrophic markers for pDEST32 and pDEST22 vector selection, respectively. To verify yeast double transformation, yeast cells carrying the vector combinations shown in the figure were grown in solid SD medium lacking both leucine and tryptophan (SD/-Leu/-Trp. Protein-protein interactions were evaluated by dropping 5 μL of overnight yeast cultures (10^6^ CFU.mL^-1^) in SD/-Leu/-Trp/-His, where histidine (His) is the auxotrophic marker for protein-protein interaction, and grown at 30°C for 3 days. To reduce background growth due to residual activity of *HIS3* gene, 3 mM of 3-Amino-1,2,4-Triazol (3AT), a competitive inhibitor of the HIS3 reporter enzyme, was added to test plates.

#### Protein isolation and western blotting

Seedling cultivation and sampling occurred as for RNA sampling. Total protein was isolated in 1.1 M glycerol, 5 M Tris-MES (pH 7.6), 1 mM EGTA and 2 mM dithiothreitol with protease inhibitor cocktail P9599 (Sigma). Protein concentrations were quantified with Bradford reagent (Bio-Rad). Proteins were separated on 10% polyacrylamide gels and transferred to nitrocellulose membranes (Bio-Rad), which were subsequently stained with Ponceau Red to verify equal protein loading. Membranes were incubated with KIN10 antiserum (AKIN10/SNF1-related protein kinase catalytic subunit alpha antibody, Agrisera) at 1:1000 dilution with 1% w/v fat-free milk powder and 0.1% v/v Tween 20, and incubated subsequently with goat anti-rabbit IgG HRP conjugate (GtxRb-003-DHRPX from ImmunoReagents, Raleigh, NC) at 1:2000 dilution. Blots were developed using Pierce ECL-2 reagent (Thermo Scientific). Two independent biological repeats were performed of each experiment (data from one repeat shown in Figure S1F).

#### Transcriptome data meta-analysis

Lists of rhythmic genes and lists of genes that are regulated by KIN10 were compared and their overlaps analyzed for significance. Rhythmic genes were selected from two nycthemeral experiments (light/dark) and three circadian experiments (constant light) [23, 24, 25, 26, 27]. Lists of genes with all the rhythmic transcripts in the nycthemeral experiments or all the circadian genes in the circadian experiments were also analyzed. The KIN10-regulated genes were selected from genes having altered expression in *KIN10*-ox [17] or in lines overexpressing the *E. coli* trehalose 6-phosphate synthase (OtsA), which elevates Tre6P *in planta* [20]. Statistical significance of overlaps and the representation factor were estimated using web-based software designed by Jim Lund (University of Kentucky), with statistical significance quantified using a hypergeometric test [61] (http://elegans.uky.edu/MA/progs/overlap_stats.html). 25% - 40% of transcripts upregulated by *KIN10*-ox and 19% - 34% of transcripts downregulated by *otsA*-ox oscillate under light/dark cycles. 49% - 59% of transcripts upregulated by *KIN10*-ox and 37% - 41% of transcripts downregulated by *otsA*-ox oscillate under constant conditions. Each overlap was calculated as the proportion of the total number of rhythmic transcripts in each phase bin. Only overlaps with p < 0.01, and representation factor < 0.5 (#) or > 2 (^∗^) and were considered significant.

### Quantification and Statistical Analysis

Statistical analysis was performed using Sigmaplot 13.0. Details of statistical tests used, replication levels, and nature of error bars are provided in figure legends. Circadian rhythm parameters were determined using the Biological Rhythms Analysis Software System (BRASS) (University of Edinburgh; millar.bio.ed.ac.uk). Statistical significance of intersections between transcriptomes was calculated with a hypergeometric test [61]. No data were excluded from analysis. Statistical significance is indicated by the p value, where ^∗^ = p < 0.05, ^∗∗^ = p < 0.01 and ^∗∗∗^ = p < 0.001.

## Supplemental Information

Document S1. Figures S1–S3 and Table S1

Document S2. Article plus Supplemental Information
