# Supplementary material for: Circadian Entrainment in Arabidopsis by the Sugar-Responsive Transcription Factor bZIP63
Source: Curr Biol. 2018 Aug 20;28(16):2597–2606.e6. doi: 10.1016/j.cub.2018.05.092 (PMC6108399; doi:10.1016/j.cub.2018.05.092)
Supplement: Document S1. Figures S1–S3 and Table S1 [file mmc1.pdf]

Current Biology, Volume *28*

Supplemental Information

Circadian Entrainment in *Arabidopsis*

by the Sugar-Responsive Transcription Factor bZIP63

Alexander Frank, Cleverson C. Matiolli, Américo J.C. Viana, Timothy J. Hearn, Jelena Kusakina, Fiona E. Belbin, David Wells Newman, Aline Yochikawa, Dora L. Cano-Ramirez, Anupama Chembath, Kester Cragg-Barber, Michael J. Haydon, Carlos T. Hotta, Michel Vincentz, Alex A.R. Webb, and Antony N. Dodd

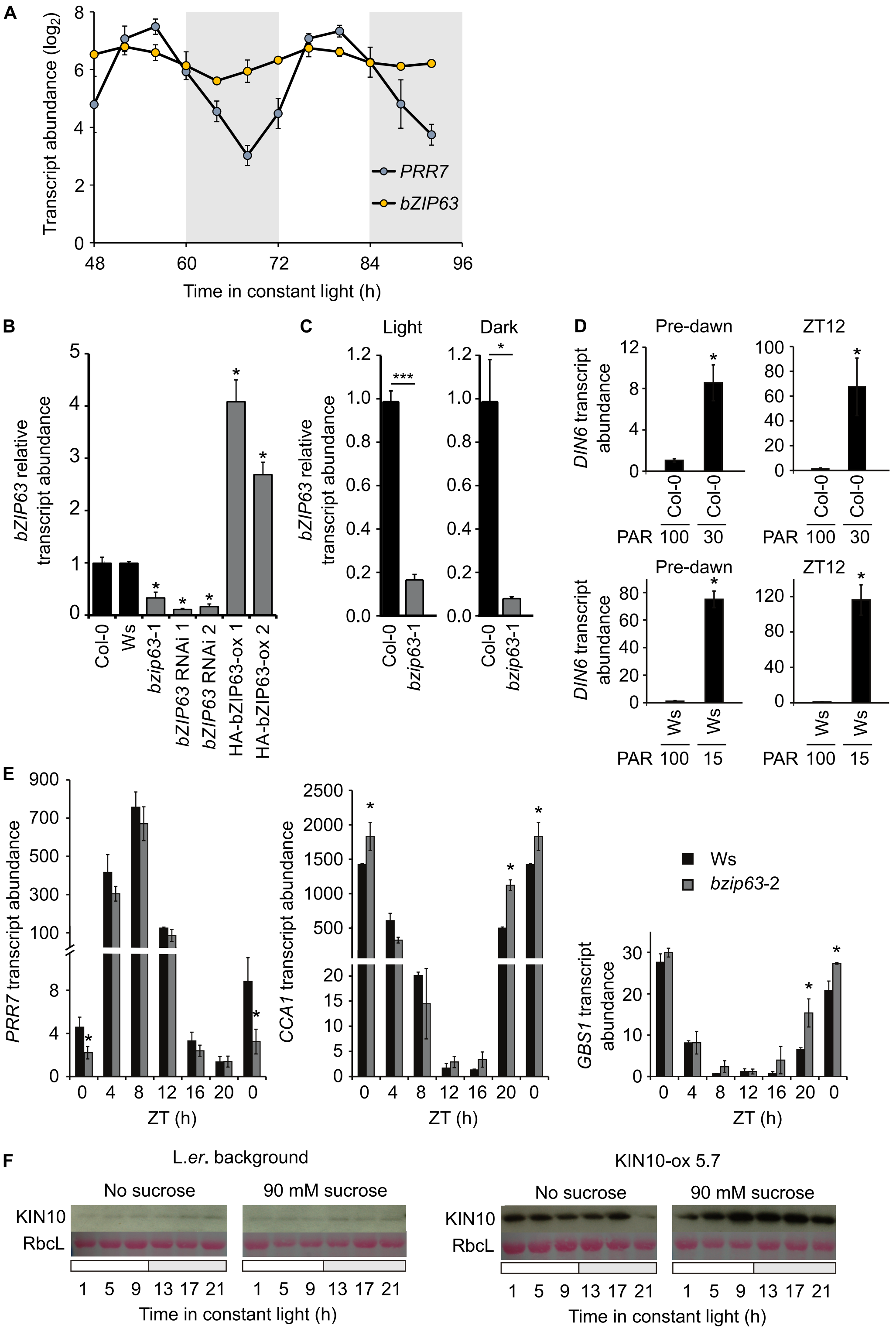

Figure S1. bZIP63 regulates *PRR7* transcript accumulation and confirmation of KIN10 overexpression. Related to Figure 1. (D) *bZIP63* transcripts have circadian oscillations in abundance that peak prior to *PRR7*. *bZIP63* transcript abundance peaked at ZT6 and *PRR7* transcript abundance peaked at ZT8 (n = 2). Data

comprise a 48 h timecourse of transcript abundance in constant light extracted from data deposited at http://www.ncbi.nlm.nih.gov/geo/query/acc.cgi?acc=GSE19271. (B) *bZIP63* transcript abundance in *bzip63* mutants and transgenic lines at ZT0. Plants were grown for 30 days under 100 μmol m^{-2} s^{-1} of cool white fluorescent light, and photoperiod of 12 h light 12 h dark. RNAi lines 1 and 2 and HA-bZIP63-ox 2 were within the Ws background, and HA-bZIP63-ox 1 was in a homozygous line harbouring the T-DNA FLAG_610A08 within the *bZIP63* coding sequence. (C) Confirmation that *bZIP63* transcript accumulation is reduced in the *bzip63*-1 mutant. Seedlings were grown in MS/2 liquid medium for 7 days under continuous 60 μmol m^{-2} s^{-1} photon flux of cool white fluorescent light, and transferred to dark for 24 hours (Dark), or maintained under light for the same conditions (Light). (D) Transcript abundance of the starvation marker *DIN6* under high and low light conditions, immediately before dawn and immediately before dusk (ZT24). Photosynthetically active radiation (PAR, μmol m^{-2} s^{-1}) indicated below graph panels. (B-D) Transcript abundance is the mean of three biological replicates (n = 3) normalized to *PP2AA3* and analyzed using Student's T-test; *** = $P < 0.001$; ** = $P < 0.01$; * = $P < 0.05$. (E) Transcript abundance of *PRR7*, *CCA1* and the direct CCA1 target *GBS1* in Ws and *bzip63*-2 plants grown for 30 days under 12 h light / 12 h dark cycles of 100 μmol m^{-2} s^{-1} white light. (F) Western blot analysis confirms KIN10 protein overexpression in *KIN10*-ox line [S1]. KIN10 protein abundance shown relative to the Rubisco large subunit band (RbcL) on the same blot stained with Ponceau red. Samples were collected and processed in parallel, with L. *er*. and *KIN10*-ox samples run on separate gels. (D, F) Grey panels on timecourses indicate subjective dark period.

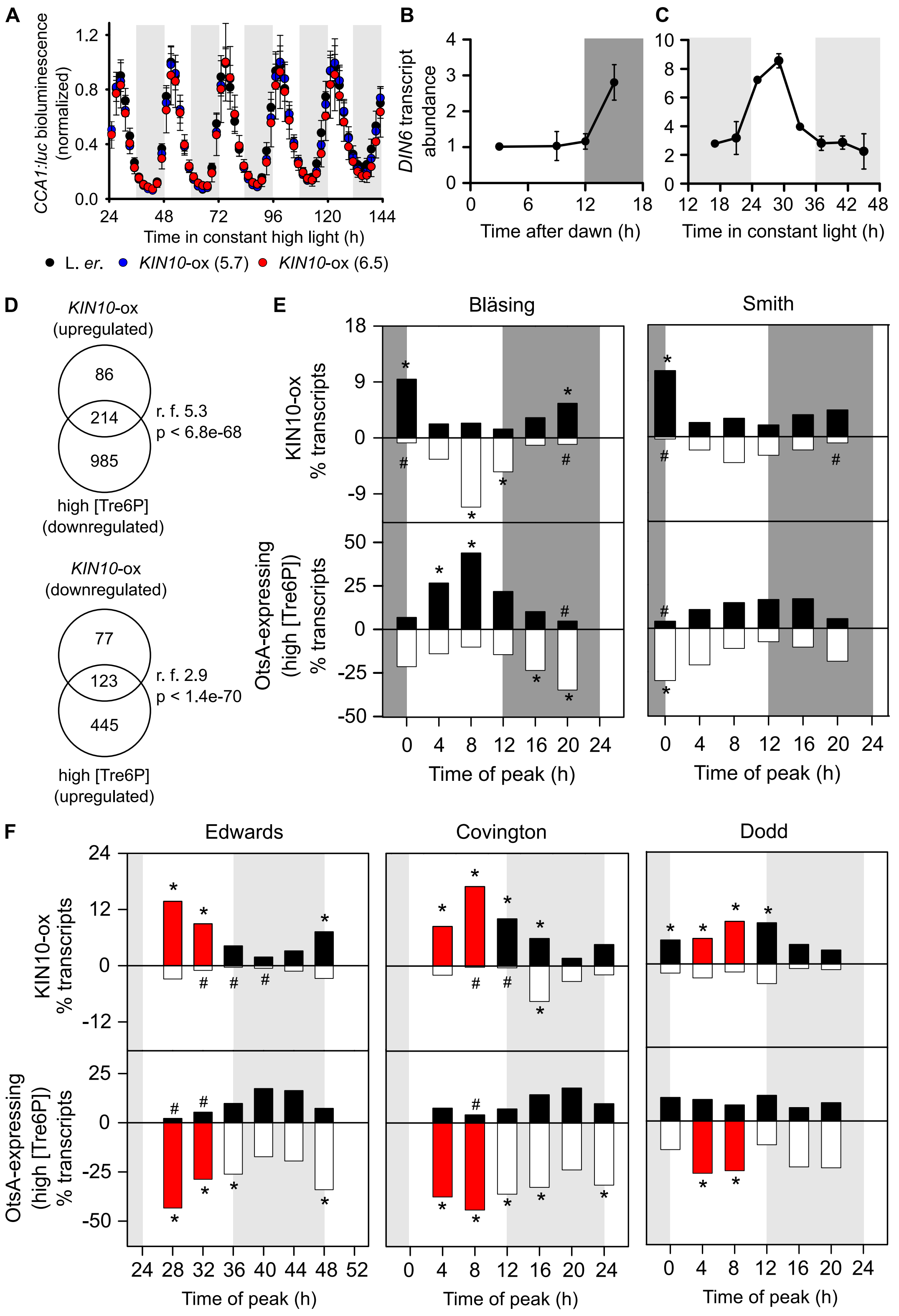

Figure S2. Interaction between circadian regulation and sugar signalling. Related to Figure 3. (A) Circadian oscillations of *CCA1:luc* activity in L. er. wild type and two constitutive KIN10 overexpressors under high light (50 μmol m^{-2} s^{-1}).

Circadian periods were L. *er*. 23.5 ± 0.1 h; KIN10-ox (5.7) 23.6 ± 0.1 h; KIN10-ox (6.5) 23.6 ± 0.1 h. n = 6 ± s.e.m. (B) *DIN6* transcript abundance in seedlings under cycles of 12 h high light / 12 h darkness. (C) *DIN6* transcript abundance under continuous light in seedlings entrained previously to cycles of 12 h light / 12 h darkness. Experiments performed without sucrose supplementation. n = 3; ± s.e.m under 50 µmol m^{-2} s^{-1} photon flux. (D-F) *In silico* analysis reveals intersection between transcripts that were altered in lines where the KIN10 pathway was misregulated [S1, S2]. (D) Statistically significant sets of transcripts are regulated by both KIN10 and Tre6P, demonstrated by intersections between transcripts upregulated by KIN10 overexpression and downregulated by high [Tre6P] (which would be predicted to suppress KIN10 activity), and transcripts downregulated by KIN10 overexpression and upregulated by high [Tre6P] (which would be predicted to increase KIN10 activity). The representation factor (r. f.) is the actual number of transcripts in the overlaps, divided by the expected number of transcripts in the overlap if the interaction were random. (E) Rhythmic transcripts identified from two light/dark experiments [S3, S4] and (F) three circadian experiments in constant light [S5-S7] that are also regulated by Tre6P/KIN10. Transcripts are plotted by phase, showing upregulated (black bars) and downregulated (white bars) transcripts. Each overlap plotted as a proportion of the total number of rhythmic transcripts in each phase bin. * indicates overlaps having more transcripts than expected than by chance, # indicates overlaps with fewer transcripts than expected by chance. Statistical significance and representation factor were estimated using a hypergeometric test [S8]. Overlaps with $P < 0.01$, and representation factor < 0.5 (#) or > 2 (*) were considered significant. Overlaps identified as statistically significant in all three circadian datasets are coloured red. In all panels, light and dark grey

shading indicates actual and subjective darkness, respectively.

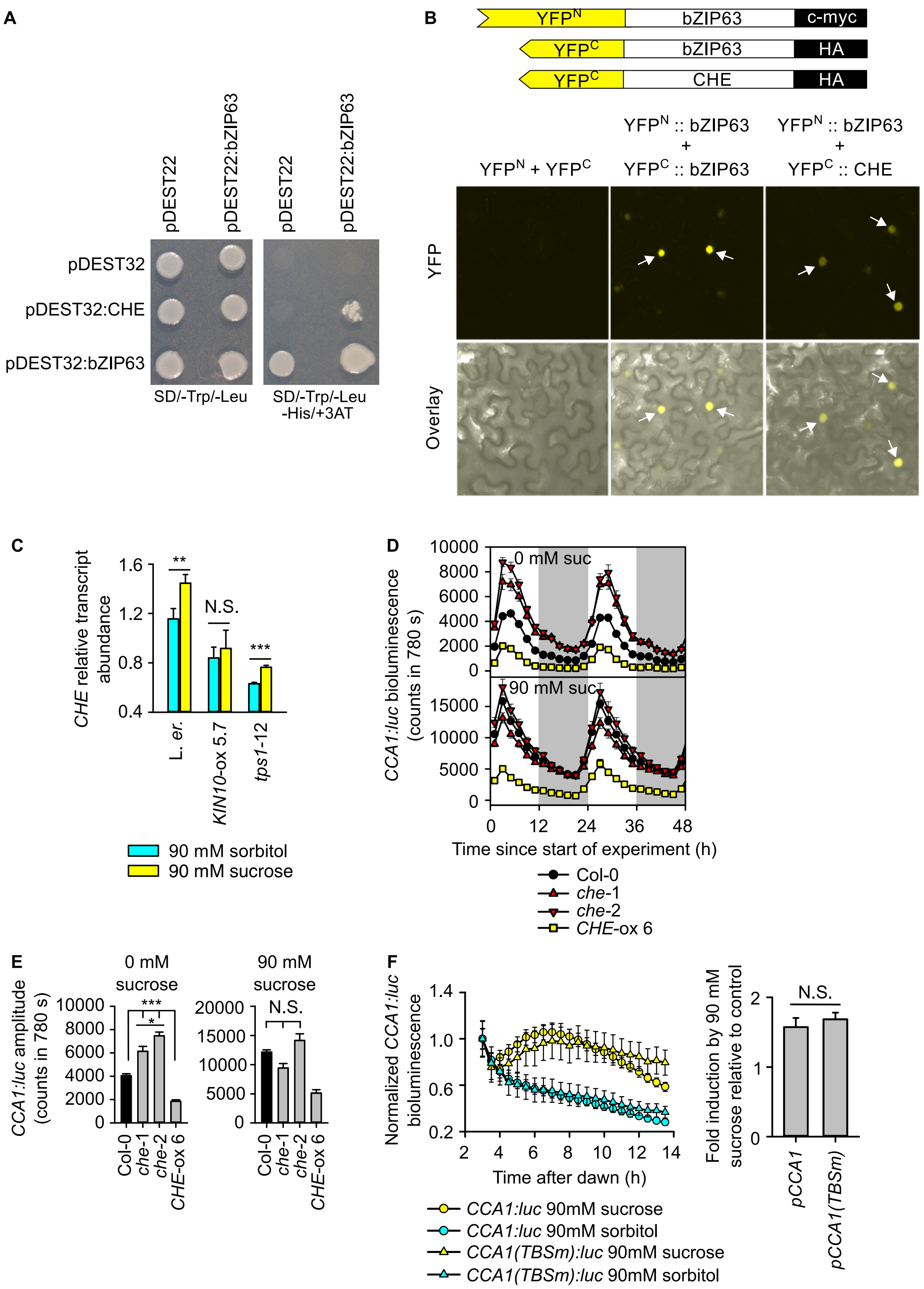

Figure S3. bZIP63 interacts with CHE and CHE regulates *CCA1* in a sucrose-dependent manner. Related to Figure 3. (A) Yeast two-hybrid (Y2H) assay demonstrating interaction between bZIP63 and CHE proteins. *bZIP63* and *CHE* full-

length coding sequence cloned in pDEST32 were used as bait, and *bZIP63* in pDEST22 as prey. The combinations between empty pDEST32 and empty pDEST22 or pDEST22:bZIP63; pDEST32:CHE or pDEST32:bZIP63 and empty pDEST22 vectors were used as negative controls for protein interaction. The combination of pDEST32:bZIP63 and pDEST22:bZIP63 was used as positive control, since bZIP63 can form strong homodimers [S9]. Yeast growth of pDEST32:bZIP63 + pDEST22 can be attributed to bZIP63 transactivation activity. (B) Confocal laser scanning microscopy images of bimolecular fluorescence complementation (BiFC) of protein fusions bZIP63-YFP^{N}, bZIP63-YFP^{C} and CHE-YFP^{C} transiently expressed in *Nicotiana benthamiana* leaves. The combinations YFP^{N} + YFP^{C} and bZIP63-YFP^{N} + bZIP63-YFP^{C} were used as negative and positive control for protein interaction, respectively (bZIP63 can form homodimers). Arrowheads indicate position of the nucleus of abaxial epidermal cells. Images were obtained three days after infiltration with *Agrobacterium tumefaciens* carrying the constructs, and are representative of at least 30 independent observations for each vector combination. (C) Response of *CHE* transcripts to 1 h pulse of 90 mM sucrose; mean from two independent experiments (*n* = 3 in each) analysed using Mann-Whitney U test. (D, E) CHE modulates the sensitivity of *CCA1* amplitude to sucrose under light/dark cycles (10 μmol m^{-2} s^{-1}), *n* = 6 +/- s.e.m. Amplitude (E) is calculated from (D) and statistical significance from one-way ANOVA and post-hoc Tukey analysis. Grey panels on timecourse indicate periods of darkness. (F) Upregulation of the *CCA1* promoter by a 90 mM sucrose pulse at ZT3, under low light conditions (10 μmol m^{-2} s^{-1} photon flux), does not require the CHE-interacting TCP transcription factor binding site (TBS) within the *CCA1* promoter. In *CCA1(TBSm):luc*, the TBS was removed from the

CCA1 promoter [S10]. Fold induction of the *CCA1* promoter, 3 h after treatment with 90 mM sucrose is indicated on right hand graph (mean from 4 independent experiments each with $n = 6$; $P = 0.535$ from t test). *** = $P < 0.001$; * = $P < 0.05$; N.S. = $P \geq 0.05$.

Primer	Sequence
Forward primer for cloning bZIP63 CDS (overexpressor)	TCTAGAATGGAAAAAGTTTTCTCC
Reverse primer for cloning bZIP63 CDS (overexpressor)	GGATCCCTACTGATCCCCAACGCT
Forward primer for cloning bZIP63 CDS (RNAi)	AAGCTTGGTACCTCACTGGTCGGTTAATGG
Reverse primer for cloning bZIP63 CDS (RNAi)	TCTAGACTCGAGCACTTGTTATAGCACTGC
Forward primer for cloning DIN6 promoter	CGTGGTACCTGGACATGAGTGCATGAC
Reverse primer for cloning DIN6 promoter	GCGAAGCTTGAAGAAAGTGAAAAAGATCACG
PRR7 qRT-PCR primer (forward)	TTCCGAAAGAAGGTACGATAC
PRR7 qRT-PCR primer (reverse)	GCTATCCTCAATGTTTTTTATGT
PP2AA3 qRT-PCR primer (forward)	CATGTTCCAAACTCTTACCTG
PP2AA3 qRT-PCR primer (reverse)	GTTCTCCACAACCGCTTGGT
CHE qRT-PCR primer (forward)	TAATGGGTGGTGGTGGTTCTG
CHE qRT-PCR primer (reverse)	TAATGGGTGGTGGTGGTTCTG

DIN6 qRT-PCR primer (forward)	TTCACCTTTCGGCCTACGAT
DIN6 qRT-PCR primer (reverse)	ATCGGCATGTTGTCAATTGC
ACT2 qRT-PCR primer (forward)	TGAGAGATTCAGATGCCCAGAA
ACT2 qRT-PCR primer (reverse)	TGGATTCCAGCAGCTTCCAT
ChIP PRR7 primer pair 1 (forward)	GACGTTTTCCTTACCCACCA
ChIP PRR7 primer pair 1 (reverse)	ATTGGCGAGGATTAGTGACG
ChIP PRR7 primer pair 2 (forward)	TGCTTTTGTATGGTTGGATTTTT
ChIP PRR7 primer pair 2 (reverse)	TGCTTTTGTATGGTTGGATTTTT
BiFC CHE cloning primer (forward)	TAAGCAGGATCCATGGCCGACAACGACGGAGC
BiFC CHE cloning primer (reverse)	TAAGCAGGTACCACGTGGTTCGTGGTCGTC
BiFC bZIP63 cloning primer (forward)	TAAGCAGGATCCATGGAAAAAGTTTTCTCCG
BiFC bZIP63 cloning primer (reverse)	TAAGCAGGTACCCTGATCCCCAACGCTTC
Y2H bZIP63 cloning primer	CACCATGGAAAAAGTTTTCTCCGAC

(forward)	
Y2H bZIP63 cloning primer (reverse)	CTACTGATCCCCAACGCTTC

Table S1: Cloning and qRT-PCR primers. Related to STAR Methods.

Supplemental References

S1. Baena-González, E., Rolland, F., Thevelein, J.M., and Sheen, J. (2007). A central integrator of transcription networks in plant stress and energy signalling. Nature *448*, 938-942.

S2. Zhang, Y., Primavesi, L.F., Jhurreea, D., Andralojc, P.J., Mitchell, R.A.C., Powers, S.J., Schluepmann, H., Delatte, T., Wingler, A., and Paul, M.J. (2009). Inhibition of SNF1-related protein kinase 1 activity and regulation of metabolic pathways by trehalose-6-phosphate. Plant Physiology *149*, 1860-1871.

S3. Smith, S.M., Fulton, D.C., Chia, T., Thorneycroft, D., Chapple, A., Dunstan, H., Hylton, C., Zeeman, S.C., and Smith, A.M. (2004). Diurnal changes in the transcriptome encoding enzymes of starch metabolism provide evidence for both transcriptional and posttranscriptional regulation of starch metabolism in *Arabidopsis* leaves. Plant Physiology *136*, 2687-2699.

S4. Bläsing, O.E., Gibon, Y., Günther, M., Höhne, M., Morcuende, R., Osuna, D., Thimm, O., Usadel, B., Scheible, W.-R., and Stitt, M. (2005). Sugars and circadian regulation make major contributions to the global regulation of diurnal gene expression in Arabidopsis. The Plant Cell *17*, 3257-3281.

S5. Dodd, A.N., Gardner, M.J., Hotta, C.T., Hubbard, K.E., Dalchau, N., Love, J., Assie, J.-M., Robertson, F.C., Jakobsen, M.K., Gonçalves, J., et al. (2007). The Arabidopsis circadian clock incorporates a cADPR-based feedback loop. Science *318*, 1789.

S6. Covington, M.F., and Harmer, S.L. (2007). The circadian clock regulates auxin signaling and responses in *Arabidopsis*. PLoS Biol *5*, e222.

S7. Edwards, K.D., Anderson, P.E., Hall, A., Salathia, N.S., Locke, J.C.W., Lynn, J.R., Straume, M., Smith, J.Q., and Millar, A.J. (2006). FLOWERING LOCUS C mediates natural variation in the high-temperature response of the Arabidopsis circadian clock. The Plant Cell *18*, 639-650.

S8. Kim, S.K., Lund, J., Kiraly, M., Duke, K., Jiang, M., Stuart, J.M., Eizinger, A., Wylie, B.N., and Davidson, G.S. (2001). A gene expression map for *Caenorhabditis elegans*. Science *293*, 2087.

S9. Ehlert, A., Weltmeier, F., Wang, X., Mayer, C.S., Smeekens, S., Vicente-Carbajosa, J., and Dröge-Laser, W. (2006). Two-hybrid protein–protein interaction analysis in Arabidopsis protoplasts: establishment of a heterodimerization map of group C and group S bZIP transcription factors. The Plant Journal *46*, 890-900.

S10. Pruneda-Paz, J.L., Breton, G., Para, A., and Kay, S.A. (2009). A functional genomics approach reveals CHE as a component of the *Arabidopsis* circadian clock. Science *323*, 1481.
